# RH knowledge and service utilization among unmarried rural-to-urban migrants in three major cities, China

**DOI:** 10.1186/1471-2458-11-74

**Published:** 2011-02-02

**Authors:** Zhiyong Liu, Minmin Zhu, Hassan H Dib, Zi Li, Shuhua Shi, Zengzhen Wang

**Affiliations:** 1 School of Public Health, Tongji Medical College, Huazhong University of Science and Technology, NO. 13 Hangkong Road, Wuhan, 430030, Hubei, PR China; 2 School of Medicine & Health Management, Tongji Medical College, Huazhong University of Science and Technology, NO. 13 Hangkong Road Wuhan, 430030, Hubei, PR China; 3 Shenzhen Nanshan Center for Chronic Diseases Control, NO. 7 Huaming Road, Nanshan District, Shenzhen, 518054, Guangdong, PR China; 4 Peking University Health Science Center, School of Public Health, Health Policy and Management, NO. 38 Xue Yuan Road, Haidian District, Beijing 100083, PR China; 5 School of Public Management, Chongqing Technology and Business University, NO. 27, Xuefu Road, Nanan District, Chongqing, 40067, PR China

## Abstract

**Background:**

Large numbers of unmarried migrants are on the continuous move from rural-to-urban areas within China mainland, meanwhile their Reproductive Health (RH) is underserved when it is compared with the present urban RH policies. The purpose of this study is to investigate the RH knowledge and the utilization of RH services among unmarried migrants.

**Methods:**

A cross-section survey was performed in three cities in China-Shenzhen, Guangzhou and Wuhan. A total of 3,450 rural-to-urban unmarried migrants were chosen according to a purposive sampling method. Around 3,412 (male: 1,680, female: 1,732) were qualified for this study. A face-to-face structured questionnaire survey was used, which focused on the knowledge concerning "fertility, contraception and STD/AIDS," as well as RH service utilization.

**Results:**

Among unmarried migrants the RH knowledge about pregnancy-fertilization (29.4%) and contraception (9.1%) was at its lowest level. Around 21% of unmarried migrants had pre-marital sexual experience and almost half (47.4%) never used condoms during sexual intercourse. The most obtained RH services was about STD/AIDS health education (female: 49.6%, male: 50.2%) and free prophylactic use of contraceptives and/or condoms (female: 42.5%, male: 48.3%). As for accessing RH checkup services it was at its lowest level among females (16.1%). Those who migrated to Shenzhen (OR = 0.64) and Guangzhou (OR = 0.53) obtained few RH consultations compared to those in Wuhan. The white collar workers received more RH consultations and checkup services than the blue collar workers (all group P < 0.01).

**Conclusion:**

RH knowledge and the utilization of RH services amongst unmarried migrants remain insufficient in the three studied major cities. This study reveals the important gaps in the RH services' delivery, and highlights the requirements for tailored interventions, including further research, to address more effectively the demands and the needs of the unmarried migrant populations.

## Background

The goal of the 1994 UN International Conference on Population and Development (ICPD) Program of Action (signed by 179 countries) is to achieve a universal accessibility for the general population towards Reproductive Health (RH) care services. This is done by mainly concentrating on providing the young people a safe, affordable, and effective RH care services as well as promoting the gender perspective [1]. Accordingly, RH care is defined as the constellation of methods, techniques and services that could contribute to the RH among the population by tackling and preventing the RH problems. RH care services also concentrates on providing better sexual health care services to enhance the quality of life and the personal relationships, rather than only consulting and caring for the related reproductive health and treating sexually transmitted diseases (STDs) [2].

As part of the family planning program in China, RH services and education are mostly conducted in the urban areas under the birth control program targeting only married women. In 2005, the UN Millennium Project report cited "the expanding access to sexual and RH information and services, including family planning" is a necessity [3]. So, from this perspective, new rules and policies in China were put forward that started to emphasize on the equality and rights of each individual to receive health education and public service irrespective of a person's place of residence [4]. Urban population refers to people who have official recognition of their resident status in the urban areas, while a floating or migrant population refers to people whose official recognition of their resident status is not in the specified urban areas, although they live and often work in the urban areas. However, the migrant workers are considered a disadvantaged population in accessing all types of working skills, work settings, social support networks and health services [5]. Studies that were conducted in other countries found that migrants are the most vulnerable group within the society in receiving health care services. This is attributed to the existence of low education among the young migrants, low awareness about HIV and/or sexually transmitted diseases (STDs), and insufficient ability of accessing and utilizing the local basic RH care services to prevent high risk behaviors such as those related to AIDS and STDs [4,6]. Young migrants are more likely to encounter problems as a result of broad changes in work and living circumstances combined with socioeconomic and cultural shock. These factors may impact their attitude towards marriage and their sexual life [4,7]. According to 2005 sampling survey performed on 1% of the population, the total number of migrants in China reached to 0.15 billion (accounted as young adults) and 71.1% of migrants are between 15-34 years old, a large proportion of which are unmarried [8]. Furthermore, as for the sexual behavior among migrants, irrespective of gender, the sexual maturity among youth is occurring at an earlier age than it was in the previous decades. Also, the sexual attitude is changing among young people; thus, indicating an increase in the premarital sexual behaviors [9,10]. In many developing countries, studies were performed on reproductive health conditions among young people, which revealed a broad lack in the necessary knowledge about sex and reproductive health as well as competence and awareness about self-protection [11-14]. Unsafe sexual behaviors have driven unmarried young migrants to expose themselves to many risks such as premarital pregnancies, induced abortion, venereal disease and STD/AIDS [15,16].

The present and existing RH care services in China are traditionally provided for the married people according to the fertility control policy. As for the unmarried group, the physical and psychological health education is provided only at schools. The unmarried young migrants, as a significant social group, who graduated or dropped out of schools, lack in the basic health education about RH services, and their RH needs are being ignored [17-19]. One study showed that the governmental family-planning workers were ambivalent about the provision of RH services to unmarried young people [20]. Due to the serious situation, the young migrants are facing several sexual behaviors and RH problems-the main concerned issue at the ICPD, requiring more attention from the governmental and non-governmental social sectors [17,21].

With the support of the international health organizations, some pilot cities in China were chosen to supply several RH services to the unmarried migrants. Since 2003, Marie Stopes international initiated "you and me" RH service centers in Qingdao, Nanjing and Nanning, and expanded to cities in Guangdong province. In general, there are limited studies on the utilization of RH services for the unmarried migrants [17,22]. From research aspect, some researchers studied the needs of RH services [19,22], others mainly concentrated on the married migrants [13,23,24] and school adolescents [25], while there are few large scale researches that have addressed any of the above issues.

Since unmarried migrants are a vulnerable group in the urban areas, we assumed that they have very limited autonomy and accessibility to RH services. Hence, to increase our understanding about the unmarried migrants' utilization of urban RH service provision, we have evaluated their knowledge and utilization of RH services. In doing so, our aim is to provide the scientific evidence that can makes us develop an appropriate planning in order to improve RH service provision for the unmarried migrants.

## Methods

A cross-sectional survey was performed from 1^st ^November 2007 to 15^th ^December 2007 in three major cities, Wuhan, Guangzhou and Shenzhen in China. The three typical cities were chosen because they have the same characteristics in housing large scale of rural-urban migrants. The proposal was reviewed and approved by the IRB of School of Public Health, Tongji Medical College of Huazhong University of Science and Technology.

### Sampling

A Purposive sampling method was used. Firstly, two streets were chosen (*recommended by the population and family planning sector in local government, naming these streets with high proportion of migrants*) from each district of the three cities and reached a total of 40 selected streets. Then, from each chosen street one community was sampled. In the last stage, all unmarried migrants (*the marriage status was recorded in local government*) in the community were recruited. The qualified migrants in the survey were without any local household registration, but lived in these communities for at least 6 months with an age range between 15-35 years and with no history of any mental diseases according to medical records in the community health centers. A total of 3,450 migrants (male: 1,700, female: 1,750) were chosen to participate in the survey, 3,420 agreed and 3,412 (male: 1,680, female: 1,732) were valid.

### Survey instrument

After our research group had performed a thorough literature review [18,26,27], consulted with several experts and applied a pre-test survey, a structured questionnaire was developed (additional file 1). The survey was validated by Cranach's alpha = 0.77, which proved the validity of the questionnaire. The questionnaire contained four parts: Part 1, contained demographic, social and economic characteristics; Part 2, contained knowledge on fertility, contraception and national family planning policy, and sex behavior. This part consisted of 13 questions which included awareness about pregnancy and contraception. The responses included: "yes" or "no", and 2 questions on the sex behavior; Part 3, STD/AIDS knowledge, which contained 18 "yes" or "no" choices of answers; we considered an answer "accurate" if a participant had correctly answered 6 or more out of 8 STD questions and 7 or more out of 10 AIDS transmission route questions; Part 4, RH service utilization, which consisted of 4 questions such as: "Have you ever obtained health education on RH", "Have you ever obtained health education on STD/AIDS prevention", and "Have you ever obtained RH checkup service" in this city; response answers included: "regularly", "few" and "never". The participant was classified as "knowledgeable" when they answered all the questions accurately.

### Data collection

This study was part of a whole project (assessment of rural-to-urban migrants RH service quality) supported by NSFC. Students from the School of Public Health of Tongji Medical College, Huazhong University of Science and Technology were recruited and trained as interviewers. All the interviewers had previous experiences in qualitative as well as quantitative data collection. At the beginning of the survey, participants were told about the aim and major contents of the survey to reduce any errors or bias as much as possible ("self protect" bias). Interviewers filled out the questionnaires and interviewed participants anonymously after the latter had provided verbal consents. If a participant failed to provide verbal consents, then the survey was terminated instantly. All the procedures were done independently face-to-face in a private room. Certain potential sensitive questions, such as sexual behavior and condom use, were held privately during the investigation.

### Definition

#### Reproductive health

within the framework of WHO's definition, reproductive health addresses the reproductive processes, functions and systems at all stages of life. Reproductive health, therefore, implies that people are able to have a responsible, satisfying and safe sexual life where they have the capability to reproduce and have the freedom to decide; if when and how often to do so [2].

#### RH consultations

consultation on contraceptive, sexual dysfunction, birth defects, reproductive tract infection, STD/AIDS, venereal consultation, prenatal care, and so on.

#### RH check

for females, it contains type B ultrasonic check, breast perspective, cervical smear, gynecological examination, etc; while for males, it contains genital inspection, semen test, and so on.

### Statistical analysis

All data were entered twice with Epi Info version 3.01 (a word processing, database, and statistics program for public health on IBM-compatible microcomputers. Centers of Disease Control and Prevention, Atlanta, Georgia, USA, 1995), using data entry screens with extensive interactive error, range, and consistent checking. After data cleaning and quality control, statistical analysis was performed using SPSS for Windows Version 12.0 (SPSS, Chicago, IL, USA). Items on utility of RH services were changed into dichotomies, i.e. answers of "regularly" and "few" were dummied as "1", "never" was dummied as "0".

Demographic characteristics, RH knowledge and behavior, and RH services' utility were analyzed descriptively; then gender or regional differences among these variables were tested using chi-square test. The effect of social and demographic characteristics on the utilization of services by unmarried migrants is studied using bivariate logistic regression analysis. Dependent variable in the analysis is a dummy variable indicating whether (1) or not (0) a migrant has received RH service since entering this city. Independent variables are socio-demographic-economic characteristics which consist of sex, age, education, located city, monthly income, and occupation. Statistical significance level (α) was set at 0.05.

## Results

### Demographic and social characteristics of Participants

Participants' education level was found to be at low level and more than half of the migrants “were below high school education level”. Most participants (58.8%) were between 20-24 years old with less than 5 years working experience. A large proportion of participants were found to be working in the manufacturing enterprises, others in trade and lastly those working in the food industry. Many of the migrants (51.1%) had an average monthly income of 600-1000 RMB (1 dollar = 6.82 RMB). Fifty-six percent was inter-provincial migration, except for Wuhan where 89.1% of the migrants had migrated within the province (See table 1).

**Table 1:** Social and demographic characteristics of unmarried rural-to-urban migrants in the three chosen cities in China

characteristics	No. of Gender (%)		Total
	**Male (n = 1680)**	**Female (n = 1732)**	**(n = 3412)**

**Ethnic**			
Han	1622 (96.6)	1648 (95.2)	3270 (95.6)
minority	56 (3.4)	84 (4.9)	140 (4.1)
**Education**			
Illiterate	14 (0.8)	20 (1.2)	34 (1.0)
Elementary school	46 (2.7)	194 (11.2)	240 (7.0)
Junior school	810 (48.2)	747 (43.1)	1557 (45.6)
high school/technical secondary school	640 (38.1)	582 (33.6)	1222 (35.8)
College and above	170 (10.1)	180 (10.9)	350 (10.3)
**Age**			
15-19	412 (24.5)	602 (34.8)	1014 (29.7)
20-24	1012 (60.3)	994 (57.4)	2006 (58.8)
25-29	198 (11.8)	110 (6.4)	308 (9.0)
≥30	58 (3.5)	26 (1.5)	84 (2.7)
**Work Experience **(years)			
Less than 1	261 (15.5)	254 (14.7)	515 (15.1)
1~	430 (25.6)	470 (27.1)	900 (26.4)
2~	312 (18.6)	338 (19.5)	650 (19.1)
3~	341 (20.3)	416 (24.0)	757 (22.2)
5~	228 (13.6)	178 (10.3)	406 (11.9)
≥10 year	108 (6.5)	76 (4.4)	184 (5.4)
**Occupation**			
construction	20 (1.2)	0	20 (0.6)
traffic and storage	28 (1.7)	0	28 (0.8)
communication	10 (0.6)	4 (0.2)	14 (0.4)
trade and food	358 (21.3)	570 (32.9)	928 (27.2)
real estate	6 (0.4)	8 (0.5)	14 (0.4)
finance and insurance	6 (0.4)	10 (0.6)	16 (0.5)
manufacturing enterprise	916 (54.5)	896 (51.7)	1812 (53.1)
social work	238 (14.2)	166 (9.6)	404 (11.8)
Self-employed ^a^	98 (5.8)	78 (4.5)	176 (5.2)
**Monthly income (RMB)**			
Under 600	86 (5.1)	66 (3.8)	152 (4.5)
600~	756 (45.0)	986 (56.9)	1742 (51.1)
1000~	490 (29.2)	440 (25.4)	930 (27.3)
1500~	192 (11.4)	188 (10.9)	380 (11.1)
above2000	156 (9.3)	52 (3.0)	208 (6.1)
**Migrated type**			
Inter province	960 (57.1)	950 (54.9)	1910 (56.0)
within province	720 (42.9)	782 (45.2)	1502 (44.0)
**City**			
Guangzhou	662 (39.4)	708 (40.9)	1370 (40.2)
Shenzhen	490 (29.2)	522 (30.1)	1012 (29.7)
Wuhan	528 (31.4)	502 (29.0)	1030 (30.2)

Note: ***a****. self-employment laborers refer to people who run a private small-scale business by themselves, such as vendors, rice-noodle sellers, food-shop owners, and tailor-shop owners.*

### RH knowledge and condom use

Table 2 shows participants' RH knowledge (pregnancy, contraception, national family planning and STD/AIDS) and their condom use behavior. Unmarried migrants demonstrated low levels of RH knowledge on pregnancy-fertilization (29.4%) and contraception (9.1%). Females' participants showed more knowledge about pregnancy, contraception and STDs, but with less knowledge on family planning policy than the male participants. Research had illustrated that males played a key role in choosing the most appropriate contraceptive methods [19], while this survey indicated that females' accessibility was higher than males. Therefore, it is of crucial importance to strengthen to improve RH services and education about contraception especially when it is targeting men. Also, participants aged 15-19 years old had the least knowledge on pregnancy and family planning policy (P < 0.001). Among the career categories, self-employed business workers showed that they had the most knowledge on pregnancy and STD/AIDS transmitted approach, while the white collar workers showed possession of good knowledge at family planning policy and STD/AIDS transmission. Furthermore, among the surveyed migrant young adults, 470 male (28.0%) and 246 female (14.2%) had pre-marital sex experiences, but only 47.4% of them used condoms, including 16.1% who used condoms half the time and only 4.0% used condoms every time. The most interesting finding is the relatively high level of knowledge about STDs among participants and nearly half of the participants received free contraceptive/condoms. However, the proportion of study participants' who actually used condoms was very low. This suggests that the use of a condom does not only depend on knowledge and availability, but that other factors play a role in the decision making process, such as perceptions of risk[28], embarrassment [29], and cultural aspects of condom use[30].

**Table 2:** Participants' distribution of responses in the three chosen cities in China about reproductive health knowledge (accurate) and condom

	RH knowledge (n, %)				Condom use (n, %)			
	
	Pregnancy and fertilization knowledge	contraception knowledge	Family planning policy knowledge	STD/AIDS spread approach	Never used	occasionally	half the time	Every time
**By sex**								
Male(1680)	403 (24.0)***	146 (8.9)	744 (44.3)	886 (52.7)	232 (49.4)	153 (32.6)	67 (14.3)	18 (3.8)
Female(1732)	601 (34.7)	161 (9.3)	705 (40.7)	948 (54.7)	107 (43.5)	80 (32.5)	48 (19.5)	11 (4.5)
**By age**								
15-20(1014)	194(19.1)	93(9.2)	355(35.0)	430(42.4)				
20-24(2006)	672(33.5)**	183(9.3)	879(43.9)**	1152(52.4)**				
25-29 (308)	103(33.5)**	30(9.7)	171(55.6)**	196(53.6)**				
30 and above(84)	36(42.6)***	6(7.1)	42(50.0)**	56(62.7)**				
**Occupation type **^*b*^								
blue-collar(2788)	800(28.7)	259(9.3)	948(34.0)	1628(53.4)				
Self-employed laborers(448)	160(35.8)*	29(6.4)	196(43.8)**	301(62.3)*				
White-collar(176)	75(42.6)**	22(12.4)	98(55.6)**	104(55.0)				
**By cities**								
Shenzhen(1012)	293(29.0)	71(7.0)	405(40.0)	588(55.1)				
Guangzhou (1370)	395(28.8)	141(10.3)	527(38.5)	747(54.5)				
Wuhan (1030)	317(30.8)	99(9.6)	521(50.6)*	499(48.4)**				

Notes: *** p ≤ 0.001; ** p ≤ 0.01; * p ≤ 0.05;

**b**. *we have regrouped "construction, traffic and storage, trade and food, manufacturing enterprise" to blue-collar and "communication, real estate, finance'' to white collar.*

### RH service obtained/utilization

The RH services usually contain RH consultations, health education on STD/AIDS, RH checkup and free condoms. The most often obtained RH services by subjects was the STD/AIDS prevention health education (female: 49.6%, male: 50.2%), and only 16.1% of females knew and took advantage of the free RH checkup (see Table 3). Among the three cities, participants in Wuhan used more RH consultations and free RH checkups; in Guangzhou and Shenzhen participants received more STD/AIDS health education, while in Shenzhen participants were the most who received free distributed contraception/condoms products. Females had more opportunities to acquire RH education than males, while males obtained more free contraception tools.

**Table 3:** RH services utilized/obtained by unmarried rural-to-urban migrants in the three chosen cities in China

Services	Shenzhen (n = 1370)	Guangzhou (n = 1012)	Wuhan (n = 1030)	all	χ2 value	P value
**Consult for RH**						
Female obtained	330 (24.1)	241 (23.8)	404 (39.2)	975 (28.3)	19.313	0.001
male obtained	444 (32.4)	205 (20.3)	330 (32.0)	979 (27.5)	14.487	0.001
**STD/AIDS preventive Health education**						
female obtained	673 (49.1)	509 (50.3)	492 (47.8)	1674 (49.6)	3.143	0.09
male obtained	704 (51.4)	528 (52.1)	474 (46.0)	1706 (50.2)	14.487	0.001
**RH checkup service **^**c**^						
free obtained	292 (9.3)	149 (14.1)	262 (25.5)	703 (16.1)	28.229	0.0001
purchased	6 (0.4)	12 (1.2)	28 (2.7)	46 (1.4)		
never use	1235 (90.2)	851 (84.1)	740 (71.9)	2826 (82.6)		
**Free contraception prophylactic or condom**						
female obtained	123 (50.0)	78 (31.6)	99 (42.5)	246 (40.4)	13.278	0.001
male obtained	247 (52.6)	185 (39.3)	227 (48.3)	470 (46.9)	13.231	0.001

Note: c. ''Pre-test results shows that males got a higher ratio of refusing to respond (more than 90%), then we set it as an item just for females.''

### The impact of social and demographic characteristics on RH service use

In order to understand the social and demographic factors related to the utilization of RH services (RH consultation, RH checkup and RH education), a logistic regression analysis was performed (See Table 4). It showed that males were poorer for RH consultation service utilization, but received more RH health education in contrast to females. Education attainment had a significant positive effect on RH consultation and RH checkup services, i.e., the higher their education level the more there is utilization of RH services. However, participants with junior and primary school education level they have obtained most of the RH health education and they were the least who had utilized the check up services. This might be an indication that the educational background plays a key role in health conceptions or health believes, and that there is a need for a long time intervention to attain behavioral change, as it is shown in the behavioral change model (e.g. the "Knowledge-awareness-behavior model"). Delivering a couple of RH health educational lectures might improve RH knowledge, but it might not automatically change peoples believes, awareness, etc; nevertheless, this might not hint there will be a change in utilizing the health care services. More 'behavioral change' strategies are needed to narrow this gap especially that can target a population with low education level. Furthermore, the white-collar workers had obtained more RH consultation and RH checkup services than heavy labor workers (blue-collar workers). Income had no statistical significant impact in the three models.

**Table 4:** Logistic regression coefficients predicting RH service utilization in three Cities in China

Variables (obtained = 1, not = 0)	RH consult services	STD/AIDS health education	RH checkup services
**Gender **(ref: female)	0.67 (0.59, 0.75)**	1.81 (1.59, 2.05) **	/
**City**(ref: Wuhan)			
Shenzhen	0.64 (0.55, 0.75)***	1.58 (1.32, 1.91)**	0.15 (0.12, 0.19)***
Guangzhou	0.53 (0.46, 0.61)***	1.24 (1.06, 1.48)**	0.26 (0.21, 0.32)***
**Education **(ref: Illiteracy)			
Elementary school	1.36 (1.18, 1.63)***	1.29 (1.23, 1.34)**	1.96(1.68, 2.28)***
Junior school	2.36 (1.95, 2.94)***	1.47 (1.14, 1.89)**	2.76 (2.37, 3.82)**
high school	3.48 (2.90, 4.31)**	0.97 (0.87, 1.33)	3.67 (2.88, 4.65)**
college and above	4.01 (2.63, 6.25)***	0.99 (0.79, 1.24)	3.70 (2.33, 5.88)**
**Age **(ref:15-19)			
20-24	1.65 (1.50, 1.78)**	1.22 (1.06, 1.42)*	1.84 (1.53, 2.06)**
25-29	3.74 (2.23, 4.03)***	1.31 (1.14, 1.56)**	2.22 (1.78, 2.57)***
>30	2.94 (2.27, 3.70)***	1.18 (0.94, 1.47)	3.92 (2.76, 5.11)***
**Work experience **(ref:<1)			
1~	1.17 (0.92, 1.49)	0.97 (0.85, 1.23)	0.63 (0.46, 0.87) ***
2~	1.01 (0.83, 1.21)	1.04 (0.86, 1.25)	0.73 (0.57, 0.93)**
3~	0.90 (0.73, 1.11)	1.28 (1.06, 1.55)**	0.94 (0.72, 1.21)
5~	1.03 (0.87, 1.23)	1.11 (0.94, 1.32)	0.93 (0.75, 1.16)
>10	1.12 (0.95, 1.32)	1.14 (0.97, 1.34)	0.84 (0.68, 1.04)
**Monthly income **(ref: <600)			
600~	1.03 (0.78, 1.34)	1.09 (0.80, 1.65)	0.90 (0.60, 1.41)
1000~	0.97 (0.79, 1.25)	1.12 (0.90, 1.54)	0.84 (0.62, 1.16)
1500~	1.08 (0.96, 1.27)	1.07 (0.86, 1.39)	0.85 (0.62, 1.20)
> = 2000	1.45 (1.02, 1.79)*	0.99 (0.80, 1.28)	0.91 (0.65, 1.22)
**Occupation **^b ^(ref: Blue-collar)			
white-collar	1.33 (1.11,1.61)***	0.67 (0.54, 0.83)**	1.47 (1.14, 1.89)**
Self-employed laborers	0.99 (0.98,1.03)	0.52 (0.45, 0.57)**	1.29 (1.23, 1.34)**
**Constant**	1.077**	3.38**	19.36***

Notes: *** p ≤ 0.001; ** p ≤ 0.01; * p ≤ 0.05;

**b**. *we have regrouped “construction, traffic and storage, trade and food, manufacturing enterprise “ to blue-collar and “communication, real estate, finance” to white collar*.

## Discussion

Though RH utilization in China had improved greatly among migrants, this study showed that the situation still holds a gloomy picture. The young unmarried migrants are comparatively receiving/utilizing less RH services, and at the same time their limited RH knowledge about pre-marital sexual contacts is jeopardizing them to higher risks of STDs.

In relation to RH knowledge, as compared to other studies [16,31], our results showed that the situation had greatly improved for the STD/AIDS information; thus, reaching highest percentage (53.8%). Our results could be correlated to the efforts of the Chinese government in focusing on AIDS education and service provision [32]. The Chinese government had drafted and implemented several policies to improve the related RH service provision. However, based on our results, RH information received by the young migrants did not attain the requirements or goals that were set up by the "China Plan of Action for Containment and Control of HIV/AIDS (2006-2010)" [33]. In this study, in comparison with the married migrants [18], the unmarried migrant population have illustrated much lower knowledge on pregnancy-fertilization (41.6% VS 14.2%) and contraception (21.3% VS 9.1%). It is shown in the previous researches that the fingerprints of the Chinese central government had left a significant impact on the birth control policy among the married migrant population, but the utilization of knowledge and methods of RH services among unmarried migrants needs further enhancement. In this study, 21.0% of the young unmarried migrants (male: 28.0%, female: 14.2%) had experienced sexual intercourse; higher than the rates of premarital sexual behavior that was previously reported: 16.0% [17], 12.2% [22], 17.6% and 8.6% [34]. These results imply that it is necessary to improve the utilization of proper contraceptive methods by adopting new effective intervention in order to avoid risky sexual behaviors. Research shows that males played a key role in choosing the most appropriate contraceptive methods [18], while this survey indicated that females' accessibility was higher than males. Therefore, it is of crucial importance to strengthen the RH services and education about contraception especially when targeting men.

Migrants' accessibility to RH services in the three typical major destination cities for migrant workers, i.e., Shenzhen, Guangzhou and Wuhan, differed from one another in many aspects. Shenzhen and Guangzhou are located in the southern coastal area containing more developed economies and majority of migrant labors. Around 75% in Shenzhen and 70% in Guangzhou are migrant labors floating from all over the country. Meanwhile, Wuhan, which is located in Central China, has a medium level inland developing economy with over one million floating population moving from many places within Hubei Province. These three cities have different migrants' needs and demands of RH services. The birth control services were the main form in Wuhan, while STD/AIDS prevention was the main form in Shenzhen and Guangzhou. Since 2003, the Population and Family Planning Commission of Guangdong Province had initiated the "AIDS prevention and family planning services, a combination of international cooperation program". In recent years, the implementation of "Publicity and Promotion of STD/AIDS Prevention Knowledge" has been accelerated in Shenzhen City and Guangzhou City. Nevertheless, the young migrant population in this study failed to use the best of the RH services that are provided by the various types of health institutions. This indicates that it is urgent to promote RH education among this group of population. Study participants with highest income (above 2000 RMB) were superior in utilizing RH services in comparison to those with lower income; similar observations were seen in previous researches [18,21].

The logistic regression model showed that the social and demographic characteristics had almost different impact on "RH consultations, RH checkup" and "STD/AIDS health education". This might be explained by the difference between the ways of "RH consultations, RH checkup" and "STD/AIDS health education" were provided. STD/AIDS health education is an active service model provided by the RH services system with more comprehensive pathways, such as using advertised posters placed in various places within the community and/or working places, through TV broadcasting, community propaganda by health professionals, and so on. On the other hand, RH consultations and RH checkup services are usually provided passively by hospitals and Maternal and Child Health Care clinics. Hence, the utilization of the RH consultations and RH checkups services usually depended on the unmarried migrants, i.e. those with high education level and those receiving more salary made more use of these kinds of services.

In recent years, China's central government policies allowed migrants to obtain an equivalent and good quality of RH services similar to the services provided to the local city residents [4,18]. However, there is absence of any well-developed RH service programs that targets unmarried migrants. The young unmarried migrant population relies on the mass media to acquire information, which in return had done little in publicizing about sex education and RH knowledge [18]. Here is the opportunity to allow more involvement of the mass media in RH education. Government departments, i.e. Family planning administration and health management should pay more attention to these matters and take some measurements and adopt new strategies in order to provide the appropriate, specific, friendly and accessible services for the unmarried young people. Such measures should focus on health education for the general population, and males specifically with lower education level. These measures could be depicted in the form of making some popular TV plays and/or Public Service Advertisements, constructing RH service evaluation system which could show the satisfaction of the unmarried rural-to-urban migrants about the services being provided.

## Conclusion

In general, we have found that RH knowledge and the utilization of RH services amongst unmarried migrants in the three major cities remains insufficient. This gap highlights the needs for tailored interventions. Hence, there is a need to perform further research and practice in order to address more effectively the needs of the unmarried migrant populations.

Further research could include in-depth studies that could investigate the RH service providers' experiences rather than only focusing on the users' side. Other recommendations that may need to be addressed as well include qualitative, in-depth interviews to gain a deeper understanding of peoples behavior and motivations, for example to use, or not use, RH services, to apply new knowledge and to make use (or not) of free contraception methods such as condoms.

## Limitations

Certain groups of the population were not included in this study, such as prostitutes, because prostitution is banned by law in China. Therefore, the pre-martial sexual activity proportion maybe underestimated. Also, there are limitations in the questionnaire due to the insufficient amount of questions to gain an in-depth understanding of the experiences and motivations behind participants' actions. Still, this paper presents valuable findings on which to build future studies.

## Competing interests

The authors declare that they have no competing interests.

## Authors' contributions

Study concept and design: LZY and WZZ. Acquisition of data: LZ. Analysis and interpretation of data: LZY, SSH, ZMM and LZ. Drafting of the manuscript: LZY and ZMM. Critical revision of the manuscript for important intellectual content: LZY, ZMM, HHD and WZZ. Statistical analysis: LZY and ZMM. Obtained funding: LZ. Administrative, technical, and material support: LZY, LZ and WZZ. Study supervision: WZZ. All authors read and approved the final manuscript.

## Pre-publication history

The pre-publication history for this paper can be accessed here:

http://www.biomedcentral.com/1471-2458/11/74/prepub

## Supplementary Material

Additional file 1 **Questionnaire for unmarried migrant RH**. The questionnaire contained four parts: Part 1, contained demographic, social and economic characteristics; Part 2, contained knowledge on fertility, contraception and national family planning policy, and sex behavior; Part 3, STD/AIDS knowledge; Part 4, RH service utilization. Click here for file
