# Supplementary material for: RH knowledge and service utilization among unmarried rural-to-urban migrants in three major cities, China
Source: BMC Public Health. 2011 Feb 2;11:74. doi: 10.1186/1471-2458-11-74 (PMC3044659; doi:10.1186/1471-2458-11-74)
Supplement: Additional file 1 — Questionnaire for unmarried migrant RH. The questionnaire contained four parts: Part 1, contained demographic, social and economic characteristics; Part 2, contained knowledge on fertility, contraception and national family planning policy, and sex behavior; Part 3, STD/AIDS knowledge; Part 4, RH service utilization. [file 1471-2458-11-74-S1.DOC]

**ID：□□□□**

**Questionnaire for unmarried migrant RH**

**Introduction:** “My name is… I’m working for… We’re interviewing people here in [name of city, region or site] in order to find out about reproductive health problems of boys and girls. Have you been interviewed in the past few weeks [or other appropriate time period] for this study? **IF THE RESPONDENT HAS BEEN** **INTERVIEWED BEFORE, DO NOT INTERVIEW THIS PERSON AGAIN.** Tell them you cannot interview them a second time, thank them, and end the interview. If they have not been interviewed before, continue:

**Confidentiality and consent:** “I’m going to ask you some questions .Your answers are completely confidential. Your name will not be written on this form, and will never be used in connection with any of the information you tell me. You do not have to answer any questions that you do not want to answer, and you may end this interview at any time you want to. However, your honest answers to these questions will help us better understand what people think, say and do about certain kinds of behaviors. We would greatly appreciate your help in responding to this survey. The survey will take about 20 minutes to ask the questions. Would you be willing to participate?”

001 QUESTIONNAIRE IDENTIFICATION NUMBER |___|___|___|

002 CITY____________________ (provide locally appropriate categories)

003 REGION__________________ (provide locally appropriate categories)

004 SITE______________________ (provide locally appropriate categories)

| **No.** | **Questions** | **Coding categories** | | **Codes** |
| --- | --- | --- | --- | --- |
| **Part I. General information** | | | | |
| Q101 | Gender (investigator filled) | ①male ②female | | □ |
| Q102 | What is your birthday? | Year  Month  (DON’T KNOW Month 99)  No Response 999999 | | □□□□□□ |
| Q103 | What is your ethnic? | ①Han ②else (please specify_____) | | □ |
| Q104 | What is the highest level of school you completed? | ①illiterate ②elementary school ③middle school ④high school / technical secondary school  ⑤college and above | | □ |
| Q105 | What is your present vocation? | ①construction ②traffic and storage ③communication ④trade and food ⑤real estate ⑥manufacturing ⑦finance and insurance ⑧social work ⑨self--employed ⑩unemployed | | □ |
| Q106 | Current address: | city district community building | |  |
| Q107 | Hometown address: | ______province ______city _______district(county) | |  |
| Q108. | When was the first time you migrated to find a job: | ____year _____month | | □□□□□□ |
| Q109. | How many years have you been working in this city: | ______years | | □□ |
| Q110. | Living condition: | ①house owned yourself  ②renting apartment  ③renting simple house  ④living in relative house ⑤temporary shelter  ⑥dormitory  ⑦else (please specify:__ ___) | | □ |
| Q111. | Monthly income: | ______RMB | | □□□□ |
| **Part II. knowledge on fertility, contraception and National Family Planning policy, and condom use** | | | |  |
| **Please response “As for a women with balanced menstrual cycle, when does the pregnancy occur?”** | | | |  |
| Q201 | Menstrual period | ①yes ②no | | □ |
| Q202 | Between the 12^th^and 16^th^ day to the next menses | ①yes ②no | | □ |
| Q203 | Several days before or after Menstruation | ①yes ②no | | □ |
| **Please response which is the emergency** **contraception method** | | | | |
| Q204 | Condom | ①yes ②no | | □ |
| Q205 | Pills | ①yes ②no | | □ |
| Q206 | Safe period | ①yes ②no | | □ |
| Q207 | IUD | ①yes ②no | | □ |
| Q208 | flush vagina | ①yes ②no | | □ |
| Q209 | Diaphragm | ①yes ②no | | □ |
| Q210 | Injectables | ①yes ②no | | □ |
| Q211 | Spermicide | ①yes ②no | | □ |
| Q212 | Withdrawal | ①yes ②no | | □ |
| Q213 | Are you aware of national family planning policy? (respondent describe and interviewer judge it according to the policy) | ①Right ② false | | □ |
| Q214 | Did you ever have sexual intercourse? **(Go to Part III** **if answered “NO”)** | ①yes ②no | | □ |
| Q215 | How often did you use the condom when you having intercourse？ | ① Never used ② occasionally ③ half of the time ④ Every time | | □ |
| **Part III** **STD/AIDS Knowledge** | | | |  |
| **Please response which is the STD** | | | |  |
| Q301 | AIDS | | ①yes ②no | □ |
| Q302 | Syphilis | | ①yes ②no | □ |
| Q303 | Genital herpes | | ①yes ②no | □ |
| Q304 | Acuteness wet wart | | ①yes ②no | □ |
| Q305 | Blame drench coccus of urethritis | | ①yes ②no | □ |
| Q306 | Contagious soft wart | | ①yes ②no | □ |
| Q307 | Vaginal moniliosis reproductive system | | ①yes ②no | □ |
| Q308 | Trichomonas vaginalis disease | | ①yes ②no | □ |
| **Please response which is the way to transmit STDs** | | | |  |
| Q309 | Sex behavior | ①yes ②no | | □ |
| Q310 | Kiss | ①yes ②no | | □ |
| Q311 | Homosexuality | ①yes ②no | | □ |
| Q312 | Blood transfusion | ①yes ②no | | □ |
| Q313 | Sharing needles when injection | ①yes ②no | | □ |
| Q314 | Having Dinner together | ①yes ②no | | □ |
| Q315 | Sharing natatorium | ①yes ②no | | □ |
| Q316 | Shaking hands or embrace | ①yes ②no | | □ |
| Q317 | Mother-to-children | ①yes ②no | | □ |
| Q318 | Mosquito biting | ①yes ②no | |  |
| **Part IV RH service utilization** | | | |  |
| Q401 | If you know there are organizations providing free contraception use | ①know ②do not know | | □ |
| Q402 | Have you ever obtain RH consulting service in this city？ | ① regularly ②few ③never | | □ |
| Q403 | Have you ever obtain free contraception use in this city？ | ① regularly ②few ③never | | □ |
| Q404 | Have you ever obtain STD/AIDs health education in this city？ | ① regularly ②few ③never | | □ |
| Q405 | If you know there are organizations providing RH check service？ | ①know ②do not know | | □ |
| Q406 | Have you ever obtain RH check service in this city？ | ① regularly ②few ③never | | □ |

The end ！

Interview date:

Checkup date:
